# Internet-Based Interventions for Problem Gambling: Scoping Review

**DOI:** 10.2196/mental.9419

**Published:** 2019-01-07

**Authors:** Mark van der Maas, Jing Shi, Tara Elton-Marshall, David C Hodgins, Sherald Sanchez, Daniela SS Lobo, Sylvia Hagopian, Nigel E Turner

**Affiliations:** 1 Institute for Mental Health Policy Research Centre for Addiction and Mental Health Toronto, ON Canada; 2 Rehabilitation Sciences Institute University of Toronto Toronto, ON Canada; 3 Dalla Lana School of Public Health University of Toronto Toronto, ON Canada; 4 Department of Epidemiology and Biostatistics Western University London, ON Canada; 5 School of Public Health and Health Systems University of Waterloo Waterloo, ON Canada; 6 Department of Psychology University of Calgary Calgary, ON Canada; 7 Campbell Family Mental Health Research Institute Centre for Addiction and Mental Health Toronto, ON Canada; 8 Department of Psychiatry University of Toronto Toronto, ON Canada; 9 Problem Gambling Institute of Ontario Centre for Addiction and Mental Health Toronto, ON Canada

**Keywords:** problem gambling, treatment, intervention

## Abstract

**Background:**

This study seeks to give an overview of academic research on internet-based interventions that are used to address problem gambling. The rate of treatment seeking has been demonstrated to be low across several research environments. This is in part because of the systemic barriers that treatment seekers face to accessing traditional face-to-face treatment. Making treatment resources for problem gambling available through the internet is one way to reduce the impact of those systemic barriers. The use of internet-based resources to address problem gambling has been growing, and a field of research evaluating it has developed as well. However, little has been done to summarize this collection of research.

**Objective:**

This study aimed to provide a scoping review of the use of internet-based interventions for problem gambling treatment and prevention to provide an understanding of the current state of the field.

**Methods:**

A scoping review was performed for 6 peer-reviewed research databases (Web of Science, PsycINFO, Cumulative Index to Nursing and Allied Health Literature, MEDLINE, Social Science Abstracts, and Scopus) and 3 gray literature databases (MedEdPortal, Proquest: Dissertations, and OpenGrey). Article inclusion criteria were as follows: published over the 10-year period of 2007 to 2017, including an intervention for problem gambling, and involving the use of internet to deliver that intervention.

**Results:**

A total of 27 articles were found that met the review criteria. Studies were found from several different areas, with particularly strong representation for Australia, New Zealand, and Scandinavia. Cognitive behavioral therapy was the most common form of internet-based intervention. Internet-based interventions were generally shown to be effective in reducing problem gambling scores and gambling behaviors. A wide range of interventions that made use of internet resources included text-based interactions with counselors and peers, automated personalized and normative feedback on gambling behaviors, and interactive cognitive behavioral therapies. A lack of diversity in samples, little comparison with face-to-face interventions, and issues of changes in the treatment dynamic are identified as areas that require further investigation.

**Conclusions:**

Internet-based interventions are a promising direction for treatment and prevention of problem gambling, particularly in reducing barriers to accessing professional help. The state of the current literature is sparse, and more research is needed for directly comparing internet-based interventions and their traditional counterparts.

## Introduction

### Background

Problem gambling can lead to serious consequences at the individual and societal levels. To limit the negative impact of problem gambling, a wide range of problem gambling interventions have been developed, although the uptake of problem gambling treatment lags behind those for substance use problems such as tobacco cessation programs [1]. Only a small proportion of those experiencing problem gambling seek professional help [2]. For example, a representative survey of Ontario residents found that only 6% of those identified as having a possible gambling problem at some point in their lives sought some kind of treatment [3]. A possible explanation for the low rates of treatment seeking is that there are several barriers that discourage those experiencing gambling-related harm from seeking professional help. In a review of the literature on treatment seeking among problem gamblers, Suurvali et al [2] found that such barriers included gamblers’ desires to handle their problems on their own, wanting to avoid the stress or stigma of being identified as a problem gambler, and practical issues surrounding treatment such as accessing treatment facilities.

One solution that has been offered to address these barriers is to increase the availability of gambling treatment options using new information technologies, interventions delivered over the internet in particular [1]. Offering treatment options over the internet can reduce barriers that potential treatment seekers may face in several ways. First, treatment options over the internet offer greater anonymity, which can help reduce the barriers associated with the stigma of treatment seeking [4]. In addition to encouraging treatment seeking, anonymity may also encourage more openness and honesty through the treatment process [5]. Treatment options delivered over the internet can also help treatment seekers overcome practical barriers associated with more traditional methods of treatment. Such barriers include, but are not limited to, distance to treatment facilities, conflicts between treatment availability and other constraints on time such as child care or paid work, cost of transportation to treatment facilities, and treatment relevant to cultural or language needs [2].

There have been several reviews of the current evidence of using internet-based resources to offer interventions in addressing problem gambling [6]. Giroux et al [7] conducted a review of the efficacy of interventions for problem alcohol use, problem substance use, and problem gambling delivered entirely through online environments. Their review found that for alcohol- and substance-focused interventions, online environments offered a great opportunity to deliver interventions that were largely similar in content and theory to those delivered through more traditional means, with the added benefit of increasing access for those that might not otherwise seek treatment. The current efficacy research shows good short-term benefits for internet-delivered interventions, although more research on long-term outcomes is needed. However, the exclusion criteria used meant no studies on gambling were included largely because of inconsistent evaluation of intervention efficacy, a focus on prevention measures and nonproblem gambling samples, and a mix of online and in-person treatment programs [7]. The paucity of research on online interventions on gambling was also identified in a review of tobacco smoking, alcohol, and gambling interventions performed by Danielsson et al [8]. However, these reviews were not focused solely on gambling and included a narrow range of study designs such as structured therapeutic interventions [6,7] or control trials [8] or failed to find gambling studies that met inclusion criteria [7]. There have also been reviews of such evidence related to other problem behaviors [9,10]. As found in a review by Barak et al, internet-based psychotherapeutic interventions show similar effect sizes (weighted mean 0.53) compared with face-to-face therapies. Their review also showed that across 14 studies, the weighted effect sizes of internet-based therapies versus face-to-face therapies were not statistically significant.

### Objective

Although delivering interventions for problem gambling over the internet has been suggested to address some of the barriers to seeking treatment for problem gambling, and several studies have shown that internet-based interventions have been shown to be effective, there is still relatively little research on the topic. The purpose of this scoping review is to provide an overview of research on problem gambling interventions that are made available through internet (hereafter referred to as internet-based interventions). Such interventions include one-on-one counseling with a mental health professional (video or voice-only conferencing, live chat, and email contact), self-help tools, peer-to-peer support, and educational tools. This scoping review was conducted to inform the development of a provincial online problem gambling treatment resource. In particular, the information provided by this review will help direct the range of interventions to implement and identify gaps in the literature for the program to contribute to the growing knowledge base surrounding the use of internet-based interventions for problem gambling. To inform this project, it is necessary to map the current literature to identify the range of interventions being offered through internet-based resources and to identify gaps in our knowledge surrounding these types of interventions. The research question was, “How are internet-based resources being used to deliver problem gambling interventions?” This review provides information on the different types of interventions that are available, the types of populations that have been exposed to these interventions, and identifies the gaps in the knowledge surrounding internet-based interventions. This review contributes to the dissemination of current knowledge on internet-based interventions and identifies possible areas for future research, given our current understandings of the potential of such intervention strategies.

## Methods

### Definitions

The structure of this scoping review is based on the methodology laid out by Arksey and O’Malley [11]. Preferred Reporting Items for Systematic Reviews and Meta-Analyses guidelines for reporting results of literature reviews were also consulted [12]. When conceptualizing internet-based interventions, we defined such interventions as *any prevention or treatment program designed to reduce the harm of problem gambling that makes use of internet resources to deliver content or resources*. Interventions using mobile apps or mobile devices were excluded in this review.

### Search Strategy

**Figure 1:**
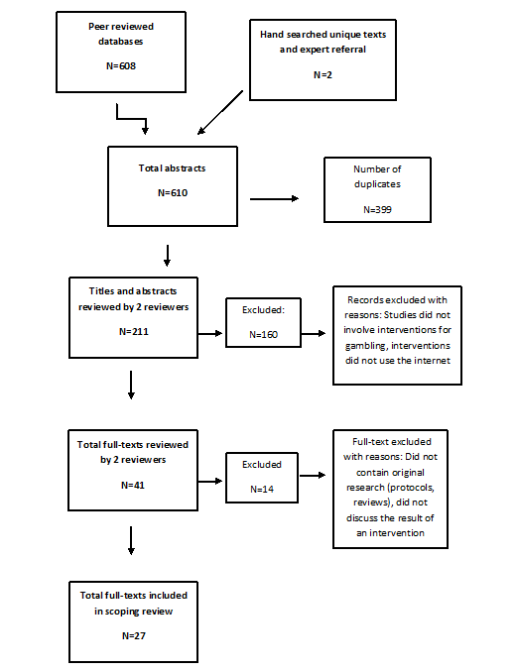
Study selection process.

Peer-reviewed journal articles were collected primarily through a search of 6 research databases: Web of Science, PsycINFO, Cumulative Index to Nursing and Allied Health Literature, MEDLINE, Social Science Abstracts, and Scopus. The gray literature was searched through the following databases: MedEdPortal, Proquest: dissertations, and OpenGrey. The following search string (modified to reflect the search logic of each database) was used to locate studies relevant to the research question: (problem* OR Patholog* OR Compuls* OR addict* OR disorder*) (adjacent within 3 words of gambl*) AND (online OR web OR internet OR internet-based OR app OR apps OR application* OR tablet* OR ipad) (adjacent within 3 words of) (therap* OR intervention* OR psychiatr* OR counsel* OR treatment*) OR (e-therap* OR etherap* OR ecounsel* OR e-counsel* OR cybercounsel* OR cyber-counsel* OR cybertherap* OR cyber-therap* OR teletherap* OR telecounsel* OR telepsychiatr*). Individual search outputs can be found in Multimedia Appendix 1. Studies were to be published between 2007 and 2017. The search strategy was designed in consultation with a team of experts in the fields of problem gambling research and treatment and with consultation with library services at the Centre for Addiction and Mental Health. Hand searches of journals especially those relevant to the field were performed. Finally, consultation with a team of experts on the research and treatment of problem gambling was performed to add articles that may have been missed using the above methods. The initial search strategy produced 610 articles. Overall, 211 articles remained after removing duplicates. The remaining article abstracts were reviewed for relevancy to the topic and to remove any publications that did not contain original research including reviews and protocol papers, leaving 41 publications. A final review of the full texts of the publications removed another 14 articles based on a lack of original research or irrelevancy to the topic, leaving a final collection of 27 articles. The process is displayed below in Figure 1.

### Study Selection, Inclusion, and Exclusion Criteria

Studies were selected for the review if they involved a primary analysis of any type of problem gambling intervention through internet or in online environments. Articles were included in this review if they involved problem gambling interventions or if they involved interventions for other substances or problem behaviors in addition to problem gambling. Such interventions included treatment, prevention, education, and early intervention. Likewise, studies were included if they involved interventions delivered solely in an online environment or if they were delivered through other media in addition to the internet.

Studies were excluded if they did not involve original research (eg, literature reviews, systematic reviews, and study protocols) and did not include information on internet-based interventions. Overall, 2 independent reviewers (1 postdoctoral fellow and 1 graduate student) reviewed all abstracts selected by the database queries. The inter-rater reliability for abstract screening was 82.5%. In cases of disagreement, a third reviewer with expertise in the field was consulted and made the final decision on inclusion. The second stage involved a review of the full-text versions of the selected articles by both reviewers. The inter-rater reliability for full-text screening was 100%.

### Data Extraction

The information from the final articles was extracted into a table that included the study aims, study sample, study and intervention design, and the central results of the study. The results of this extraction process were then synthesized and interpreted in consultation with a team of experts in the field of problem gambling treatment in Canada.

## Results

### Location

Multimedia Appendix 2 provides descriptions of the selected articles. Many of the studies involved gambling help websites that were available over the internet and as such could have been accessed by anyone with an internet connection. Due to this lack of a physical geographical location, each study will be defined as the country from which the internet-based interventions were delivered. There was a strong representation of research from Australia and New Zealand, with 7 articles examining online-delivered problem gambling interventions [13-20]. The majority of these studies were analyses of the interventions offered through *Gambling Help Online* [15-19,21]. This site has been in operation since 2009 and provides 24-hour chat and email counseling and support services, access to professional counselors, access to face-to-face or telephone counseling, and a variety of self-help resources. Several studies were also based in Europe, the majority in Scandinavian countries (namely Norway [22], Finland [23,24], and Sweden [25,26]), although there were also studies conducted in France [27], Italy [28], Germany [29], and the United Kingdom [30]. Several studies have been conducted in Canada and the United States. Ontario was the sole province included in the results [31-33], 2 American studies involved college counseling websites across America [34,35], 1 study involved participants from Nevada and Massachusetts [36], and 1 study involved undergraduate students in Oklahoma [36]. Overall, 1 study involved an international comparison of problem gambling resources available on college and university counseling websites in the United Kingdom and the United States [34].

### Sample Populations

There was relatively little range in the sample characteristics found in the studies. Many of the studies drew their samples from clients of existing gambling help websites [13-19,20-24,37]. For these studies, participants accessed the internet-based inventions voluntarily and gave consent for their information to be used for research purposes. Media advertising was also used by several studies [20,25,26,31], and 1 study surveyed grade 9 students from a single high school in Italy [28].

Most of the studies had samples with more males than females ranging from 50.6% [19] to 90.4% [30]. Overall, 2 studies used exclusively female samples [24,31]. There was also a single case study involving a woman aged 31 years [13]. Studies tended to focus on adult samples (>18 years), ranging in mean ages of 31.9 [26] to 56 [31]. Overall, 1 study included minors in its sample of grade 9 high school students [28]. The majority of studies did not explore differences in terms of cultural backgrounds. However, 1 study did explore Asian self-identification as a factor impacting concerned significant others of problem gamblers [14].

Samples of help seekers were common in the selected studies being the focus of 12 of the included studies [13,15-19,20-24,37]. As a result of recruiting participants directly from those seeking information or help problem gambling, the studies tended to have high proportions of problem or pathological gamblers ranging from 60.6% [32] to 100% [16,20] in those studies where a gambling screen was applied.

### Use of Technology

Several types of internet-based technologies were employed in the selected studies. The most common form of technology used was email contact. This was found in 10 of the selected studies [18,25-28,32,33,36-38]. Email was commonly used for feedback that did not need to be communicated in real time. This included feedback on work completed through a therapy program [25,26,28], normative or personalized feedback on gambling behaviors [32,33,36,38], and therapist contact [18,37]. Text communication was also common in real-time chat apps [14,15,18,19,21,24,31,37] and moderated discussion boards [22,23,24,26,31]. Electronic versions of digital workbooks for therapy programs were common, particularly in studies that used cognitive behavioral therapy (CBT) or Motivational Interviewing (MI) [20,22,23,25,26,27,28,31]. Other uses of internet-based resources included voice and video chat [13,31,37], pop-up messages [29], monitoring and screening services [16,38], and Web-based educational resources [34,35].

### Study Design

The goal of 2 studies was to give a profile of the consumers accessing internet-based interventions for problem gambling. Statistical analyses of those accessing online problem gambling websites were common for these studies. These studies provided descriptive profiles of users [32] and gender comparisons in the types of online resources accessed [18].

Several studies involved longitudinal designs that compared pre-and postintervention score on a variety of measures. Overall, 6 studies employed a randomized controlled trial (RCT) to test the effectiveness of internet-based interventions [20,26-28]. In each case, the comparison was made between the use of internet-based interventions versus no intervention in the control group. Overall, 1 study included another comparison treatment group including internet delivered CBT, internet delivered Monitoring Feedback and Support therapy, and a waiting list control [20]. Overall, 5 other studies employed pre/postintervention designs but without control groups [33]. Follow-up points for all studies involving pre-and postintervention assessments ranged from 1 week to 4 years with 3 to 6 months being the most common.

Overall, 8 studies employed qualitative analysis to explore the use of online resources [13,15-17,19,21,24,37]. Overall, 2 studies conducted analyses of online chat sessions or discussion boards on gambling help websites [15,24]. Other qualitative studies included typed responses to open-ended surveys delivered online, [19] and a case report of a 31-year-old woman’s experience with internet-based exposure therapy [13].

In terms of assessment and data collection, most studies used only online resources to collect information. Overall, 3 studies had some degree of face-to-face contact in addition to online resources. In addition, 1 study [13] used face-to-face assessment of gambling problems, whereas 2 studies [31,37] made at least some portion of their assessment of problem gambling over phone. For all other studies, problem gambling was assessed using online resources. Moreover, 1 study compared results from an open-label parallel-group trial with random assignment with the results of an earlier study with parallel therapy design delivered through face-to-face contact [20].

Numerous screens were employed to identify problem gambling. Specific problem gambling screens included the Problem Gambling Severity Index (PGSI) [16,17,21,27,31-33], Gambling Attitudes Scale [28], South Oakes Gambling Screen (SOGS) [13,20], SOGS-R (revised) [22], and SOGS-RA (revised adolescent) [28], and the National Opinion Research Center DSM Screen for Gambling Problems (NODS) [23,25,26]. Scales related to gambling behavior included Problem Gambling Significant Other Impact Scale (PG-SOIS) [14], Gambling Urges Scale [20], Gambling Refusal Self-Efficacy Scale [20], Gambling Symptom Assessment Scale [20], and the Diagnostic and Statistical Manual for Mental Disorders criteria for pathological gambling (DSM IV [31] and DSM V [20]). Of these scales, PGSI and NODS were the most commonly used scales. There were also other gambling-related assessments made including time and money spent on gambling, faulty cognitions, and types of gambling.

Several studies also included measures of mental health issues that are often found to be comorbid with problem gambling. These included the following: Work and Social Adjustment [13]; Beck Depression Inventory [13]; Beck Anxiety Inventory [13]; Perceived Stress Scale [31]; Depression, Anxiety and Stress Scale [20,31]; Alcohol Use Disorders Identification Test [20,23,25,26]; Montgomery-Åsberg Depression Rating Scale [23,25,26]; Hospital Anxiety and Depression Scale [23,25,26]; Quality of Life Inventory [11,20,22-26]; Satisfaction with Life Questionnaire [20]; and The Positive and Negative Affect Schedule [17].

### Types of Interventions

Several types of interventions were delivered through online resources. The most common form of intervention found was one-on-one counseling with a trained therapist [13,14,15,16,17,18,26]. These sessions were performed using a variety of methods including videoconferencing, telephone, email, and chat. Typed communications were also used in the included studies. Several studies analyzed the transcripts from chat sessions between consumers and mental health professionals on gambling help websites [14,15,17,18]. These single chat sessions were often on a nonappointment basis, and were frequently accessed by first time help seekers. For example, 1 study [15] found that 62.4% of chat session users were new to counseling. In addition to counseling targeting potential problem gamblers, 1 study focused on internet-delivered counseling for concerned significant others and explored the use of a new assessment scale for the concerned significant others of problem gamblers (PG-SOIS) [14].

CBT and other work assignment based therapies were commonly used in the included studies [20,23,26-28,31,39]. CBT programs ranged from 3 weeks [28] to 3 months [22,31]. Weekly feedback was provided to clients and took the form of either telephone or voice-only contact with a counselor or therapist or in the form of weekly email contact. Assignments and workbooks were made available through online communication.

Several studies explored the use of the internet to host a group discussion with multiple clients and mental health professionals [24,25,31,40]. The group discussion either took place in online chat spaces with simultaneous use by several clients, by mental health professional–moderated discussion boards [24], or by the use of webinars with a mental health professional facilitator [31]. These group discussions were often used in conjunction with CBT.

Although the majority of studies focused on treatment-based interventions, there were several studies that focused on prevention and early intervention strategies. These included pop-up messages [29], online responsible gambling tools [30,32,36,37,38], and problem gambling education materials [34,35].

Some forms of interventions were less common among the included studies. Overall, 1 study tested the effectiveness of a normative feedback generated based on a short survey of gambling-related activities and demographic information [29,32,33]. The goal of this intervention is to compare the participants’ gambling activities with those of similar backgrounds to motivate treatment seeking or re-education about gambling involvement. Another research team looked at the availability of gambling relevant information on college counseling websites rather than the effectiveness of interventions [22,35] and a single study used exposure therapy [13].

Several studies examined the use of the internet to deliver information-based interventions to gambling participants through the use of personalized or personalized normative feedback [27,28,29,32,33,36]. For these interventions, the data tracking possibilities offered through online gambling website allows the flagging of problem behaviors and/or delivery of targeted information related to one’s own gambling in comparison with others. This is a more efficient manner of identifying possible problem behavior than relying on help seeking or identification of problem behaviors by gambling venue staff.

### Central Findings

The majority of studies with treatment designs noted significant improvements in problem gambling over time using a variety of measures. Of the 7 RCT design studies, 5 found significant improvement from the internet-based intervention group over controls (no treatment in all cases) [20,24,26,28]. In addition to problem gambling improvement (based on problem gambling scores), these studies also found improvements in gambling behaviors, anxiety, and depression [26]. Significant improvements in problem gambling, gambling frequency [23,25], faulty cognitions surrounding gambling [22], alcohol consumption [23], and distress [17,22] were also noted in intervention studies that did not include a control group.

One study with an RCT design found that those receiving an internet-based CBT intervention did not show significant improvement in problem gambling scores compared with a control group. The authors note that this may have been because of recruiting players from an online casino website and that these participants were not seeking help [27]. Another RCT design study found that normative feedback did not offer significantly different reductions in gambling behaviors compared with controls [36]. For those studies that included treatment programs, high rates of attrition were identified. For those studies that reported them [20,22,23,27,31], attrition rates ranged from 38% [22] to 83% [27].

Several of the studies in the review identified important diversity in the ways that clients use internet-based interventions ([16,17, [38]). Use of online resources to address problem gambling was shown to be related to perceived ability and desire to change ([16,21]; greater problem gambling website usage being related to greater experience of gambling-related harm [38]).

## Discussion

### Principal Findings

The purpose of this review was to provide an overview of how internet-based resources were used in interventions for the treatment and prevention of problem gambling. The selected studies showed a wide range in the types of interventions that were being offered through internet-based resources. Most commonly, information technologies were used to modify or extend existing, popular forms of treatment for problem gambling. The most common therapy type was CBT, which was used in 6 of the 27 included studies. Other therapies included MI, Monitoring Feedback and Support, and exposure therapy. By and large, these interventions showed significant reductions in problem gambling scores and indicators of gambling involvement including time and money spent. The majority of the selected interventions (15/27) involved using the internet in some way to connect clients to mental health professionals for some kind of counseling, typically through typed chat or video sessions. This increase in access was identified as one of the key features that the internet can offer to the treatment of problem gambling [14,15,18].

Another common way that internet-based resources were used in the selected studies was using large amounts of collected data to improve the detection of potential problems or to allow potential participants to contextualize their own gambling behaviors. The ability to collect and use data from online gambling or treatment environments allows gambling providers and responsible gambling site operators to improve their harm prevention strategies efficiently [30,33].

There was a relatively small range of samples found in the review given the relatively few studies selected. Help-seeking samples were the most common. This is a result of many studies drawing their samples from clientele of problem gambling help websites. As help seeking is relatively rare among problem gamblers, it is difficult to say how representative the results are of the existing literature of the experiences of the problem gambling population. Those studies that targeted females using internet-based interventions and found that females were highly receptive to them [13,24,31]. Internet-facilitated treatment makes it possible to create single gender discussion and treatment groups. This can be especially important for females who may feel more comfortable in female-only groups but are too spread out geographically for in-person discussion groups [31]. Single-gender groups may also be important to females and males as gendered interpretations of stigma associated with problem gambling have been shown to have different effects in discouraging treatment-seeking [41].

In the selected studies, little consideration was given to the impact of age in the use of internet-based technologies in the treatment or prevention of problem gambling. Only 1 study [28] focused analysis on a sample of adolescents, whereas all other studies included adult samples with little consideration for variation in experience by age. This is unfortunately as younger clients and online gamblers were also identified as groups that were especially receptive to internet-based interventions [18]. The 1 study that used a sample of adolescents also suggested that internet-based interventions would be an effective tool in preventing problem gambling in younger cohorts. This is encouraging considering rates of problem gambling are disproportionately higher in younger cohorts and that adolescents are generally unaware of how to recognize problem gambling or how to access help [42]. However, problem gambling information available to younger cohorts is sparse [35], demonstrating that although internet-based interventions may be effective for targeting this priority population, they are currently underutilized.

### Gaps and Challenges

The included studies also show numerous challenges in using internet-based interventions. The authors of the selected studies identified a wide range of challenges and concerns associated with using internet-based technologies in the treatment and prevention of problem gambling. One of the most important challenges was a high rate of attrition as noted in several studies. However, although attrition rates were found to be high, these studies noted that they were similar to those found in studies of face-to-face interventions [1,23,31]. As noted in a review of internet-based treatments for psychological conditions, it is difficult to compare dropout rates of in-person and internet-based interventions because of inconsistent definitions and tracking of dropouts [43]. It has also been suggested that attrition rates for studies of gambling help websites may be inflated because consumers may register for gambling help sites to simply see what kinds of services are available but are not ready to use those services [1]. The convenience of internet-based interventions may also contribute to the lack of program completion and online counseling or self-help programs may only be engaged in for as long as the consumer feels they are necessary [26]. Overall, 1 study compared the results of an internet-based CBT program and found a substantially higher dropout rate (47.7%) compared with a similarly structured face-to-face program (18.6%) [20].

Another gap regarding internet-based interventions is determining whether there is deficit in rapport when compared with face-to-face interactions with mental health professionals. Rapport is an important component of effective treatment from both the perspective of the health professional and the client. For example, in a study of stated preferences and the acceptability of internet-based treatment of anxiety and depression, 71.1% of health professionals and 58.0% of lay respondents stated that they would prefer in-person treatment compared with therapy over the internet (3.9% and 9.1%, respectively) [44]. Aspects of interpersonal communication such as facial expressions and body language can be important tools for counselors in detecting distress in their clients [45,46]. Similarly, several studies that screened for problem gambling used online self-reported versions of problem gambling screeners; however, this increases the chances of diagnostic inaccuracies relative to face-to-face screening [26,32]. Although the greater anonymity offered by online resources can increase accessibility for some consumers, it can also present difficulties in tracking the progress of clients. It is possible for clients to have multiple concurrent accounts, delete old accounts, and create new accounts in the case of relapse, creating confusion in the data produced by their participation. This potential issue points to the importance of clear instructions regarding research integrity to prevent this from happening.

Internet-based interventions for problem gambling are relatively new; therefore, several studies identified a need to replicate their findings or extend their studies to new groups and therapies. In particular, there is a lack of comparison between in-person interventions and internet-based interventions. Although multiple RCT studies confirmed internet-based interventions led to significantly better improvements than no intervention (with 2 exceptions), no peer-reviewed studies examined the comparative effectiveness between online and in-person delivered treatment. The experience of treatment using online resources may be substantially different from more traditional intervention. For example, Rodda et al [19] found that the flexibility, anonymity, and style of communication (written) were important motivating factors in consumers choosing internet-based interventions over traditional face-to-face therapy. They note that although the goal of traditional helpline interventions is to ultimately direct potential consumers to face-to-face counseling, the convenience and range of intervention options available through gambling help websites makes it more likely to be the first and last source of support that they might access. Although no study directly compared the effectiveness of internet-based interventions with face-to-face interventions, 1 study did compare effect size of their internet-based intervention with the results of a previous, similarly structured study on face-to-face intervention. The results suggested that internet-based CBT delivered comparable reductions in gambling amount, gambling frequency, and improved gambling refusal efficacy. However, lower dropout rates (18.6% vs 47.7%) and lower faulty gambling cognitions were observed in the face-to-face program [20].

### Limitations

There are several limitations of this study. First, the scoping nature of this review was intended for the purpose of mapping the current literature regarding internet-based interventions for problem gambling. This means that the search strategy was not as exhaustive as may be included in a systematic review and thus was likely to miss a number of relevant articles on the subject. As noted by Arksey and O’Malley [11], the scoping nature of this review brings with it limitations. Specifically, this review is not able to assess the quality of evidence or provide an analytical synthesis of the evidence. Another limitation is the lack of inclusion of mobile device–delivered interventions (often referred to as mHealth interventions). Although many of the technologies and challenges involved in these forms of interventions may be similar, we regarded these technologies as being outside of the scope of this study. Although non-English language studies were not excluded purposefully from this review, all searches were performed in English and as such were likely to miss studies in other languages. This biases the current results to reflect predominately English language research. The search strategy was also limited as a result of focusing on search terms related to problem gambling specifically and not on more general language of gambling-related harm. As a result, the current selection strategy may be biased toward including studies focused on treatment rather than harm reduction and prevention studies. It should be noted that in some cases, several of the articles included in this review were based on data from a single program of work. Overall, 7 studies were based on data collected from *Gambling Help Online*, an Australian online counseling and support website [14-19]. This commonality between these studies should be kept in mind by the reader as it can potentially bias the findings of this study.

### Conclusions

This scoping review sought answers to 1 central question surrounding internet-based interventions: “How are internet-based resources being used to deliver problem gambling interventions?” The selected studies show that internet-based resources are primarily used to modify existing popular therapies for problem gambling, largely to increase access and flexibility. The existing body of knowledge suggests that internet-based interventions show potential but that their effectiveness compared with in-person treatment is unknown, and possible unintended side effects are largely unexplored. Researchers have found evidence that a variety of forms of internet-based interventions show positive results in treating problem gambling. However, this scoping review found a lack of replication of study or intervention designs as well as a lack of research on marginalized groups for whom barriers to access traditional treatment are the greatest. In short, although the initial research on internet-based interventions is supportive of greater deployment, there are still many unanswered questions regarding the positive and negative aspects of internet-based interventions relative to face-to-face treatment for problem gambling.

## Appendix

Multimedia Appendix 1 Search strings.

Multimedia Appendix 2 Included studies.

## Abbreviations

CBT: cognitive behavioral therapy
DSM: Diagnostic and Statistical Manual for Mental Disorders
MI: Motivational Interviewing
NODS: National Opinion Research Center DSM Screen for Gambling Problems
PGSI: Problem Gambling Severity Index
PG-SOIS: Problem Gambling Significant Other Impact Scale
RCT: randomized controlled trial
SOGS: South Oakes Gambling Screen
