# Supplementary material for: Internet-Based Interventions for Problem Gambling: Scoping Review
Source: JMIR Ment Health. 2019 Jan 7;6(1):e65. doi: 10.2196/mental.9419 (PMC6329421; doi:10.2196/mental.9419)
Supplement: Multimedia Appendix 1 [file mental_v6i1e65_app1.pdf]

Appendix I: Search strings

PsychInfo: 81 and MEDLINE: 36

1. exp gambling/

2. ((problem* or Patholog* or Compuls* or addict*) adj3 gambl*).mp.

3. or/1-2 [gambling concept]

4. online therapy/

5. ((online or web or internet or internet-based or app or apps or application* or tablet* or ipad) adj3 (therap* or intervention* or psychiatr* or counsel* or treatment*)).mp.

6. (e-therap* or etherap* or ecounsel* or e-counsel* or cybercounsel* or cyber-counsel* or cybertherap* or cyber-therap* or teletherap* or telecounsel* or telepsychiatr*).mp.

7. or/4-6 [etherapy concept]

8. 3 and 7

CINAHL: 8

MH “gambling” AND

(((problem* or Patholog* or Compuls* or addict*) n3 gambl*))

AND

((((online or web or internet or internet-based or app or apps or application* or tablet* or ipad) n3 (therap* or intervention* or psychiatr* or counsel* or treatment*)))

OR

(e-therap* or etherap* or ecounsel* or e-counsel* or cybercounsel* or cyber-counsel* or cybertherap* or cyber-therap* or teletherap* or telecounsel* or telepsychiatr*))))

Social science abstracts: 13

(((problem* or Patholog* or Compuls* or addict*) n3 gambl*).) AND (((online or web or internet or internet-based or app or apps or application or tablet* or ipad) n3 (therap* or intervention* or psychiatr* or counsel* or treatment)))

Scopus 220

(TITLE-ABS-KEY (((problem* OR patholog* OR compuls* OR addict*) W/3 gambl*) .)) AND ((((online OR web OR internet OR internet-based OR app OR apps OR application OR tablet *) W/3 (therap* OR intervention* OR psychiatr* OR counsel* OR treatment*)))) AND (LIMI T-TO (PUBYEAR , 2017 -2007)

Web of science 250

#1: TS=((problem* OR Patholog* OR Compuls* OR addict* OR disorder*) NEAR/3 gambl*)
#2 TS= ((online OR web OR internet OR internet-based OR ape OR apps OR application OR tablet* OR pad) AND (therapy* OR intervention* OR psychiatry* OR counsel* OR treatment*))

#1 AND #2
