# Supplementary material for: Internet-Based Interventions for Problem Gambling: Scoping Review
Source: JMIR Ment Health. 2019 Jan 7;6(1):e65. doi: 10.2196/mental.9419 (PMC6329421; doi:10.2196/mental.9419)
Supplement: Multimedia Appendix 2 [file mental_v6i1e65_app2.pdf]

Table 1. Included studies.

Citation	Aims	Sample	Design	Results
Auer and Griffiths, 2015 [30]	To investigate the effects of normative and self-appraisal feedback in a slot machine pop-up message compared with a simple (nonenhanced) pop-up message in an online gambling environment.	N=1.6 million in game sessions for online slot machines; country: Germany; mean age: N/A[a]; % male: N/A; %PG[b]: N/A	Comparison of the effectiveness of enhanced (normative and self-appraisal feedback) versus simple pop-up messages in ending long slots play sessions (+1000). The data were collected from the play records of a large commercial online gambling operator (bwin.party).	Including normative and self-appraisal content of pop-up messages during slots play increased the number of players who quit after receiving the message (1.39% vs 0.67%). Results show that although enhanced pop-up messages might be more effective in encouraging players to quit long play sessions, they still only influence a very small proportion of participants overall.
Boughton et al, 2016 [32]	To test a Web and telephone-based group, online intervention for women problem gamblers	N=25; country: Canada; mean age: 56 years; % male: 0; % PG: 83 (DSM IV[c])	Pilot study of a weekly webinar with workbook modules. MI[d]-based 12-week program. Teleconference/Web-based group discussion moderated by clinician.	Improvements were noted in stress and anxiety scales. Women-only therapy groups can be facilitated by the use of the internet.
Canale et al, 2016 [29]	To test the efficacy of a Web-based gambling intervention (CBT[e]) for high school–aged students.	N=168 grade 9 students; country: Italy; mean age: 15.01 years; % male: 58; % PG: N/A	A 2-month follow-up cluster randomized trial of a Web-based intervention based on CBT and MI models. The intervention involved screening, personalized feedback, and an online training program.	Intervention group showed significant improvements over time among frequent gamblers in the study (weekly or more frequent) in problem gambling scores and gambling frequency.
Carlbring	To investigate	N=284; country:	Noncomparative single group	Improvements were identified at the

et al, 2012 [26]	whether online treatment was effective for those experiencing depression and whether pretreatment characteristics were predictive of treatment success.	Sweden; mean age: 32.2 years; % male: 81%; % PG: 100 (NODS[f])	study with follow-ups at 3, 6, 18, and 36 months. CBT model with workbooks and weekly therapist contact. Digital workbooks were delivered online and feedback was given through email. Weekly telephone contact with clinician was included.	immediate time frame all the way to the 36-month follow-up. The intervention had similar effect for those who were more severely depressed. Predictive model showed it is possible to estimate success of internet-based interventions pretreatment.
Carlbring and Smit, 2008 [27]	To test if the internet-based CBT intervention was more effective than no treatment. (waiting list)	N=66; country: Sweden; mean age: 31.9 years; % male: 94; % PG: 100 (NODS)	Randomized control study of a CBT program. Digital workbooks were completed online with feedback delivered through email, and online discussion groups were used. Weekly telephone contact with clinician. Follow-ups at 3,6,18, and 36 months. Control group received no treatment.	Significant reductions were found in anxiety, depression, and problem gambling measures that were sustained at follow-up.
Casey, 2017 [21]	To investigate the efficacy of an internet-based CBT program for the treatment of problem gambling.	N=174; country: Australia; mean age: 44.3 years; % male: 41; % PG: 100 (DSM V)	Open-label parallel-group trial with random assignment to 3 conditions (internet-delivered CBT, internet-delivered monitoring feedback and support therapy, and waitlist). 6-week treatment period with 3, 6, and 12 month follow-ups. Online	Internet-based CBT produced better outcomes in several areas when compared with an internet-based Monitoring Feedback and Support intervention. Both online interventions produced significant improvements when compared with the waitlist. When compared with face-to-face interventions, internet-based interventions

			treatment activities used with telephone contact at assessment.	produced similar improvements in several areas but had a higher rate of attrition and were less effective in improving gambling-related cognitions.
Castren et al, 2013 [24,42]	To share practical experience from the field. To explore the impacts of the offered therapy for gambling-related problems, levels of alcohol consumption, and depression.	N=471; country: Finland; mean age: 34.5 years; % male: 96; % PG: 64 (NODS)	Internet-based cognitive behavioral program completed over 8 weeks. Digital work submitted online with online discussion groups. 6- and 12-month follow-ups.	Significantly reduced gambling-related problems, gambling urge, and impaired control of gambling mood and handle over social situations improved along with a decrease in alcohol consumption following treatment.
Cunningham et al, 2011 [33]	To run a pilot study of the “Check Your Gambling” screener.	N=870; country: Canada; mean age: 38.1 years; % male: 62.4; % PG: 77.9 (PGSI[g])	Pilot study of a personalized feedback program. Feedback based on demographic and gambling involvement.	Descriptive statistics of those accessing the Check Your Gambling screen for their own gambling and comparing across the 2 sites that offer it.
Dowling et al, 2014 [14]	To describe the characteristics of concerned significant others and to identify the impacts that gambling has on them.	N=366 concerned significant others; country: Australia; mean age: N/A; % male: 16.4; % PG: N/A	Qualitative analysis of nonscheduled online text-based counseling sessions. Examining the different factors (negative effects) that are associated with their gambling significant others.	Family members reported greater impact as a result of a significant other in comparison with friends. Emotional distress and impacts on relationships were the more commonly reported impacts.
Forsström et al, 2016 [39]	To identify subclasses of users of a responsible	N=9293 users; country: Sweden; mean age: 41.41 years (men),	Analysis of data collected from a large online gambling environment (Playscan).	Although initial use was high, repeated use of most functions was low. Overall, 5 different classes of users were identified

	gambling tool.	43.94 years (women); % male: 84.2; % PG: N/A	Responsible gambling tools include risk assessment, communication of risk, and strategies to decrease gambling.	based on responsible gambling tool use patterns. Those who were at higher risk of problem gambling were more likely to use the self-testing function and to be multifunction users.
Gray et al, 2017 [31]	To compare the behavioral characteristics of subscribers who triggered corporate responsible gambling interventions against controls.	N=4132; country: United Kingdom; mean age: 28.95 years; % male: 90.4; % PG: N/A	Comparison of the gambling behaviors of clients who did and did not trigger online corporate responsible gambling interventions.	Involvement in both time and money spent on gambling activities were strong predictors of whether clients would trigger corporate responsible gambling interventions. Gamblers who triggered interventions actually showed lower proportionate losses in comparison with controls. Online gambling sites provide resources for identifying and providing immediate interventions for potential problem gamblers that are less available in land-based gambling environments.
Hopper, 2008 [37]	To determine whether an electronic intervention based on normative feedback will lead to decreases in gambling behaviors over time.	N: 68; country: United States; mean age: 21.4 years; % male: 90; % PG: N/A	Analysis of personalized feedback prepared that compared the respondents' gambling behaviors with those found in their demographic groups.	No improvements were noted over time in the quantity or frequency in gambling at follow-up compared with control group. Experimental group did show decreased perceived norms in gambling.

Jarvinen-Tassopoulos, 2016 [25]	To explore the importance of familial connection on the experience of problem gambling and substance use for women.	N=574 discussion board messages; country: Finland; Mean age: N/A; % male: N/A; % PG: N/A	Qualitative analysis of online discussion board messages and online text-based counseling sessions from a problem gambling website.	Experiences of gambling-related harm were related to poor coping skills, stressful events, and difficulties in relationships. Online forms offer a good forum for introduction to counseling or help that allows the gambler to avoid stigma. In this case, the ways that the problem gambling label negatively affects their identities as mother and wives.
Luquiens et al, 2016 [28]	To determine the efficacy of 3 internet-based psychotherapies among a sample of online poker players.	N=2563; country: France; mean age: 33.49 years; % male: 92; % PG: 100 (PGSI≥5)	Internet-based RCT[h]. Statistical comparison on personalized feedback on problem gambling scores through email, CBT workbook with no guidance, the same therapy program with weekly guidance, and feedback including email and telephone contact.	Treatment conditions did not show improvements over control condition (waiting list)
McKinley et al, 2016 [35]	To determine whether problem gambling information on college counseling websites increased over the study period (2008-2013). To determine whether there are differences between United Kingdom and United	N=264; colleges with counseling center websites; country: United Kingdom and the United States; mean age: N/A; % male: N/A; % PG: N/A	Comparisons were made on information and services available online between gambling and other addiction/substance use problems.	Less than 11% of college counseling websites mentioned problem gambling. UK sites had more info than the US sites and only pamphlets became more available over the 5-year period.

	States.			
McKinley and Wright, 2012 [36]	To describe the availability of gambling-related information on college counseling websites.	N=203 colleges with counseling center websites; country: United Kingdom; mean age: N/A; % male: N/A; % PG: N/A	Description of gambling-related information and services compared with a series of other mental health issues.	Only 15% of college counseling websites had any information on problem gambling.
Myrseth et al, 2013 [23]	To determine whether clinical improvements could be measured in an online delivery of CBT program.	N=112; country: Norway; mean age: 36.99 years; % male: 87.5; % PG: 100 (SOGS-R[i])	CBT program with 9 assignments submitted online and weekly telephone interviews. Online discussion boards were made available, but not required	Significant improvements in problem gambling were noted at follow-up. Improvements also noted in distress.
Oakes et al, 2008 [13]	To demonstrate the effectiveness of videoconferencing in the use of exposure therapy.	N=1; country: Australia; age: 31 years; % Male: 0; % PG: 100 (DSM IV)	Exposure therapy with internet-delivered video counseling.	Videoconferencing was considered to be effective for both the client and the therapist. Rapport was easy to establish and the client felt comfortable.
Rodda et al, 2015 [15]	To describe the concerns of clients presenting to an immediate Web-based counseling service.	N=85; country: Australia; mean age: N/A; % male: 50.6; % PG: N/A	Thematic analysis of text-based counseling session transcripts from a gambling help website.	Clients tended to spend more time describing their past experiences with problem gambling rather than discussing strategies to mitigate gambling-related harm. Online environments may be particularly useful for those seeking immediate help.
Rodda et al, 2015 [16]	To identify different categories of problem gambler among those seeking	N=1204; country: Australia; mean age: N/A; % male: 63.7; % PG: 99.4 (PGSI)	Analysis of a questionnaire provided before online counseling session.	The study identifies 4 distinct categories of gamblers seeking help from online resources. Subtypes varied in terms of readiness to change, the importance of

	treatment over Web-based platform.			change, and confidence to resist the urge to gamble.
Rodda et al, 2017 [17]	To determine the immediate impact of online counseling sessions.	N=229; country: Canada; mean age: N/A; % male: 57; % PG: 100 (PGSI)	Analysis of pre- and postonline text counseling session distress scores.	Immediate decreases in distress levels following chat sessions were observed.
Rodda and Lubman, 2014 [18]	To describe the characteristics of the visitors of a large online gambling help website and the services they use.	N=2869; country: Australia; mean age: 34.5 years; % male: 59.6; % PG: N/A	Comparison of men and women across type of service used (online chat and email).	Chat clients were younger and male. No significant differences between email and chat in terms of ethnicity or gambling preferences.
Rodda et al, 2013 [19]	To understand the motivations for choosing Web-based help support over face-to-face.	N=233; country: Australia; mean age: N/A; % male: 60.6; % PG: 92	Qualitative analysis of open-ended survey question delivered online. Sample included those who had access to online text-based counseling.	Convenience, anonymity, flexibility, and preference for written communication were all given as motivations for online versus face-to-face. Availability of support resources made possible by online treatment options could change the nature of counseling, making it possible to provide immediate intervention instead of skills for future avoidance of problems or gambling episodes.
Rodda et al, 2017 [22]	To identify ways to influence gambling behaviors and investigate challenges in the initiation and choice	N=149; country: Australia; mean age: N/A; % male: 52.3; % PG: N/A	Thematic analysis of online text-based counseling transcripts	Overall, 6 strategies to implement change in gambling behaviors were identified. Although many of these strategies were similar to those found in research on land-based gambling, several novel actions related to the use of internet were identified. 4 issues

	of strategies to address problem gambling.			related to implementation of self-help strategies were also identified.
Wohl et al, 2017 [34]	To test the utility of a responsible gambling tool that provides players with personalized behavioral feedback about their play.	N: 449; country: Canada; mean age: N/A; % male: 59.4; % PG: N/A	Personalized feedback emailed to participants based on their perceived and recorded gambling habits. Surveys administered at baseline and 3-month follow-up periods.	Those players who had underestimated their losses showed significantly lower expenditures and losses at the 3-month follow-up, despite reporting not changing their gambling habits. Using online resources to provide gamblers with accurate information on their behaviors can help gambling participants moderate their spending.
Wood and Griffiths, 2007 [38]	To evaluate the experience of GamAid site	N=413; country: Canada; mean age: 36 years; % male: 71; % PG: N/A	Descriptive statistics based on data from gambling help website. Qualitative response to online survey for website clients included.	Majority of clients were satisfied with the site. Online gamblers and women seemed particularly pleased with the service.

[a]N/A: not applicable.

[b]PG: problem gambling.

[c]DSM: Diagnostic and Statistical Manual for Mental Disorders.

[d]MI: Motivational Interviewing.

[e]CBT: cognitive behavioral therapy.

[f]NODS: National Opinion Research Center DSM Screen for Gambling Problems.

[g]PGSI: Problem Gambling Severity Index.

[h]RCT: randomized controlled trial.

[i]SOGS-R: South Oakes Gambling Screen-R.

30. Auer MM, Griffiths MD. Testing normative and self-appraisal feedback in an online slot-machine pop-up in a real-world setting. Frontiers in psychology. 2015;6:339.

32. Boughton RR, Jindani F, Turner NE. Group treatment for women gamblers using Web, teleconference and workbook: effectiveness pilot. International journal of mental health and addiction. 2016;14(6):1074-95.

29. Canale N, Vieno A, Griffiths MD, Marino C, Chieco F, Disperati F, et al. The efficacy of a web-based gambling intervention program for high school students: A preliminary randomized study. Computers in Human Behavior. 2016;55:946-54.

26. Carlbring P, Degerman N, Jonsson J, Andersson G. Internet-based treatment of pathological gambling with a three-year follow-up. Cognitive Behaviour Therapy. 2012;41(4):321-34.

27. Carlbring P, Smit F. Randomized trial of internet-delivered self-help with telephone support for pathological gamblers. Journal of Consulting and Clinical Psychology. 2008;76(6):1090.

21. Casey LM, Oei TP, Raylu N, Horrigan K, Day J, Ireland M, et al. Internet-based delivery of cognitive behaviour therapy compared to monitoring, feedback and support for problem gambling: A randomised controlled trial. Journal of gambling studies. 2017;33(3):993-1010.

24. Castren S, Lahti T. Experiences from the field: Interned-based CBT intervention for Finnish gamblers. Psykologia. 2014;49(3):198-210.

33. Cunningham JA, Hodgins DC, Toneatto T. Pilot study of an Internet-based personalized feedback intervention for problem gamblers. Journal of Gambling Issues. 2011:3-10.

14. Dowling NA, Rodda SN, Lubman DI, Jackson AC. The impacts of problem gambling on concerned significant others accessing web-based counselling. Addictive Behaviors. 2014;39(8):1253-7.

39. Forsström D, Hesser H, Carlbring P. Usage of a responsible gambling tool: a descriptive analysis and latent class analysis of user behavior. Journal of gambling studies. 2016;32(3):889-904.

31. Gray HM, LaPlante DA, Shaffer HJ. Behavioral characteristics of Internet gamblers who trigger corporate responsible gambling interventions. Psychology of Addictive Behaviors. 2012;26(3):527.

37. Hopper RA. A brief electronic personalized normative feedback intervention for the prevention of problematic gambling among college students: Oklahoma State University; 2008.

25. Järvinen-Tassopoulos J. Problem gambling and drinking among Finnish women. Nordic Studies on Alcohol and Drugs. 2016;33(1):27-42.

28. Luquiens A, Tanguy M-L, Lagadec M, Benyamina A, Aubin H-J, Reynaud M. The efficacy of three modalities of Internet-based psychotherapy for non-treatment-seeking online problem gamblers: A randomized controlled trial. Journal of Medical Internet Research. 2016;18(2):1-13.

35. McKinley CJ, Luo Y, Wright PJ, Kraus A. Problem gambling messages on college counseling center websites: An over-time and cross-country comparison. Journal of gambling studies. 2016;32(1):307-25.

36. McKinley CJ, Wright PJ. Examining the presence of problem gambling awareness messages on college counseling center websites. Health communication. 2012;27(1):98-106.

23. Myrseth H, Brunborg GS, Eidem M, Pallesen S. Description and pre-post evaluation of a telephone and internet based treatment programme for pathological gambling in Norway: A pilot study. International Gambling Studies. 2013;13(2):205-20.

13. Oakes J, Battersby MW, Pols RG, Cromarty P. Exposure therapy for problem gambling via videoconferencing: A case report. Journal of Gambling Studies. 2008;24(1):107-18.

15. Rodda S, Lubman D, Cheetham A, Dowling N, Jackson A. Single session web-based counselling: A thematic analysis of content from the perspective of the client. British Journal of Guidance & Counselling. 2015;43(1):117-30.

16. Rodda S, Lubman D, Iyer R, Gao C, Dowling N. Subtyping based on readiness and confidence: The identification of help-seeking profiles for gamblers accessing web-based counselling. Addiction. 2015;110(3):494-501.

17. Rodda S, Lubman D, Jackson A, Dowling N. Improved outcomes following a single session web-based intervention for problem gambling. Journal of Gambling Studies. 2017;33(1):283-99.

18. Rodda S, Lubman DI. Characteristics of Gamblers Using a National Online Counselling Service for Problem Gambling. Journal of Gambling Studies. 2014;30(2):277-89.

19. Rodda S, Lubman DI, Dowling NA, Bough A, Jackson AC. Web-based counseling for problem gambling: Exploring motivations and recommendations. Journal of Medical Internet Research. 2013;15(5):162-71.

22. Rodda SN, Hing N, Hodgins DC, Cheetham A, Dickins M, Lubman DI. Change strategies and associated implementation challenges: An analysis of online counselling sessions. Journal of gambling studies. 2017;33(3):955-73.

34. Wohl MJ, Davis CG, Hollingshead SJ. How much have you won or lost? Personalized behavioral feedback about gambling expenditures regulates play. Computers in Human Behavior. 2017;70:437-45.

38. Wood RT, Griffiths MD. Online guidance, advice, and support for problem gamblers and concerned relatives and friends: An evaluation of the GamAid pilot service. British Journal of Guidance & Counselling. 2007;35(4):373-89.
